# Pulmonary infection after cardiopulmonary bypass surgery in children: a risk estimation model in China

**DOI:** 10.1186/s13019-021-01450-w

**Published:** 2021-04-07

**Authors:** Chunnian Ren, Chun Wu, Zhengxia Pan, Quan Wang, Yonggang Li

**Affiliations:** 1 Department of Cardiothoracic Surgery, Children’s Hospital of Chongqing Medical University, No.136, Zhongshan 2nd Road, Yuzhong Dis, Chongqing, 400014 P.R. China; 2 Ministry of Education Key Laboratory of Child Development and Disorders; National Clinical Research Center for Child Health and Disorders (Chongqing); China International Science and Technology Cooperation base of Child Development and Critical Disorders, Chongqing, P.R. China; 3 Chongqing Key Laboratory of Pediatrics, Chongqing Medical University, Chongqing, P. R. China

**Keywords:** Congenital heart disease, Surgery, Pulmonary infection, Risk prediction model

## Abstract

**Objectives:**

The occurrence of pulmonary infection after congenital heart disease (CHD) surgery can lead to significant increases in intensive care in cardiac intensive care unit (CICU) retention time, medical expenses, and risk of death risk. We hypothesized that patients with a high risk of pulmonary infection could be screened out as early after surgery. Hence, we developed and validated the first risk prediction model to verify our hypothesis.

**Methods:**

Patients who underwent CHD surgery from October 2012 to December 2017 in the Children’s Hospital of Chongqing Medical University were included in the development group, while patients who underwent CHD surgery from December 2017 to October 2018 were included in the validation group. The independent risk factors associated with pulmonary infection following CHD surgery were screened using univariable and multivariable logistic regression analyses. The corresponding nomogram prediction model was constructed according to the regression coefficients. Model discrimination was evaluated by the area under the receiver operating characteristic curve (ROC) (AUC), and model calibration was conducted with the Hosmer-Lemeshow test.

**Results:**

The univariate and multivariate logistic regression analyses identified the following six independent risk factors of pulmonary infection after cardiac surgery: age, weight, preoperative hospital stay, risk-adjusted classification for congenital heart surgery (RACHS)-1 score, cardiopulmonary bypass time and intraoperative blood transfusion. We established an individualized prediction model of pulmonary infection following cardiopulmonary bypass surgery for CHD in children. The model displayed accuracy and reliability and was evaluated by discrimination and calibration analyses. The AUCs for the development and validation groups were 0.900 and 0.908, respectively, and the *P*-values of the calibration tests were 0.999 and 0.452 respectively. Therefore, the predicted probability of the model was consistent with the actual probability.

**Conclusions:**

Identified the independent risk factors of pulmonary infection after cardiopulmonary bypass surgery. An individualized prediction model was developed to evaluate the pulmonary infection of patients after surgery. For high-risk patients, after surgery, targeted interventions can reduce the risk of pulmonary infection.

## Introduction

Congenital heart disease (CHD) is the most common birth defect worldwide [1].Most CHD patients require surgical treatment. Hospital-acquired infections (HAIs) are a major cause of morbidity and mortality in paediatric patients undergoing cardiac surgery [2, 3]. Pulmonary infection results in significant morbidity (e.g., increased antibiotic usage, prolonged hospital and intensive care unit (ICU) stays, and prolonged periods of mechanical ventilation and inotropic support), which contributes to an increase in mortality [2, 4]. With the progress in cardiothoracic surgery, cardiopulmonary bypass surgery, myocardial protection and postoperative nursing practices, the survival rate of children with CHD has significantly improved. However, secondary ischaemia-reperfusion injury, hypothermia and surgical trauma can induce supplementary cascade reactions, the release of endotoxins, the activation of leukocytes and vascular endothelial cells, and the release of inflammatory cytokines, leading to a temporary immunosuppressive state [5, 6]. During the process of postoperative recovery, this immunosuppressive state leaves children vulnerable to infection and increases the risk of HAIs [2].

There are reports of risk factors for nosocomial infection after CHD surgery locally and abroad, but there are few reports on pulmonary infection [2, 7, 8]. The occurrence of pulmonary infection varied among different studies; from 11.6% in hospitals in the U.S.A. to 30 to 60 and 65.6% in hospitals in Spain and Mexico,respectively [2, 3]. Pulmonary infection is a common hospital-acquired infection in children after cardiopulmonary bypass surgery; at present, we can routinely use advanced antibiotics / antiviral drugs / strong airway management for all patients after cardiopulmonary bypass surgery, but this is not in accordance with the principle of antibiotic use or the economic benefits of patients. Identifying risk factors for pulmonary infection after cardiopulmonary bypass surgery and attempting to use these risk factors to create a risk prediction model is necessary. Therefore, we established a risk prediction model based on the possibility of postoperative pulmonary infection in patients undergoing CHD surgery. We constructed a nomogram to allow doctors to screen for high-risk patients after surgery. These patients should receive targeted interventions to reduce the risk of pulmonary infections, and these interventions should be evaluated in future clinical trials. The reported risk prediction model was developed and reported in accordance with the guideline for the Transparent Reporting of a Multivariable Prediction Model for Individual Prognosis or Diagnosis (TRIPOD) [9, 10].

## Methods

### Source of data and participants

Retrospective data were collected from the Children’s Hospital of Chongqing Medical University from October 2012 to October 2018. The study was conducted in accordance with the Declaration of Helsinki and was approved by the Ethical Committee of Chongqing Medical University. Written informed consent was obtained from the patients’ parents before inclusion in this study. The research data were anonymized, and personal identifiers were completely removed.

The clinical data of 1776 patients who underwent cardiac surgery under cardiopulmonary bypass between October 2012 and December 2017 were collected. Patients admitted to the thoracic surgery ward with a clinical and two-dimensional echocardiogram-based diagnosis of CHD [11], which was established by paediatric cardiologists, and who underwent cardiac surgery under cardiopulmonary bypass during hospitalization were enrolled. No therapeutic antibiotics were administered the week before surgery. All patients entered the CICU for further treatment after surgery.

All patients entered the CICU after operation, all patients were treated separately nurse, avoiding contact with the family and the outside world. The primary end point of the analysis was “pulmonary infection,” The criteria for the diagnosis of pulmonary infection was as follows: Chest radiograph showing new or worsening lung infiltrates (cannot be explained by other reasons), coughing up purulent sputum or purulent secretions in the trachea, and having any of the following: ①Bronchoalveolar lavage fluid (BALF) or Anti-pollution brush sampling is positive for quantitative culture; ②lower respiratory tract culture and blood culture are both positive and the same pathogen; ③lower respiratory tract culture and pleural fluid culture are both positive and the same pathogen [12, 13]. For patients with incomplete demographic data, patients with preoperative pulmonary infection or patients with other disease and those without cardiopulmonary bypass surgery were excluded.

### Outcome and predictors

The primary outcome of the study was the occurrence of pulmonary infection after cardiopulmonary bypass surgery. Because our goal was to predict the probability of the occurrence of pulmonary infection after surgery by creating a model, the endpoint was the end of surgery, and the analysis included preoperative data and intraoperative data.

We selected candidate variables based on previously identified risk factors for pulmonary infection after cardiac surgery [2, 7, 8, 14] as well as potential risk factors based on the clinical suspicion of the authors. We collected and analysed the following factors for all the subjects: general information (sex, age, weight), preoperative data (risk-adjusted classification for congenital heart surgery (RACHS)-1 scores, pulmonary hypertension, preoperative hospital stay, preoperative total protein content, preoperative albumin), and intraoperative data (anaesthesia time, cardiopulmonary bypass time, aortic occlusion time, operation time, intraoperative blood transfusion). Sex, pulmonary hypertension, and intraoperative blood transfusion were set as binary variables. Age, weight, preoperative hospital stay, anaesthesia time, cardiopulmonary bypass time and aortic occlusion time, operation time, preoperative total protein content and preoperative albumin were grouped by optimal scale regression grouping (Table 1). The patients were divided into two groups according to their RACHS-1 [15–17] scores and the methods of a previous study [18].

**Table 1:** Baseline characteristics in the development group and validation group

Variable (%)	Development group(***n*** = 1776)			Validation group(***n*** = 395)		
	PI (***n*** = 580)	non-PI (***n*** = 1196)	***P*** Value	PI (***n*** = 128)	non-PI (***n*** = 267)	***P*** Value
**Sex**			**0.782**			**0.624**
**Male**	**312(53.8)**	**635 (53.1)**		**70 (54.7)**	**139 (52.1)**	
**Female**	**268 (46.2)**	**561 (46.9)**		**58 (45.3)**	**128 (47.9)**	
**Age (month)**			**<.001**			**<.001**
**< 6**	**110 (19.0)**	**65 (5.4)**		**24 (18.7)**	**10 (3.8)**	
**6–11.99**	**187 (32.2)**	**185 (15.5)**		**34 (26.6)**	**46 (17.2)**	
**12–23.99**	**154 (26.6)**	**271 (22.7)**		**48 (37.5)**	**70 (26.2)**	
**> = 24**	**129 (22.2)**	**675 (56.4)**		**22 (17.2)**	**141 (52.8)**	
**Weight (kg)**			**<.001**			**<.001**
**< 5**	**63 (10.9)**	**21 (1.8)**		**17 (13.3)**	**6 (2.3)**	
**5–7.99**	**231 (39.8)**	**240 (20.0)**		**55 (43.0)**	**54 (20.2)**	
**8–13.99**	**230 (39.7)**	**520 (43.5)**		**50 (39.1)**	**134 (50.2)**	
**> = 14**	**56 (9.6)**	**415 (34.7)**		**6 (4.6)**	**73 (27.3)**	
**PAH**			**0.251**			**0.965**
**YES**	**249 (42.9)**	**548 (45.8)**		**51 (39.8)**	**107 (40.1)**	
**NO**	**331 (57.1)**	**648 (54.2)**		**77 (60.2)**	**160 (59.9)**	
**RACHS-1**			**<.001**			**<.001**
**<=2**	**280 (48.3)**	**1106 (92.5)**		**56 (43.8)**	**256 (95.9)**	
**> = 3**	**300 (51.7)**	**90 (7.5)**		**72 (56.2)**	**11 (4.1)**	
**Preoperative hospital stay (min)**			**<.001**			**<.001**
**< 125**	**156 (26.9)**	**643 (53.8)**		**31 (24.2)**	**132 (49.4)**	
**125–174.99**	**179 (30.9)**	**365 (30.5)**		**44 (34.4)**	**78 (29.2)**	
**175–224.99**	**89 (15.3)**	**115 (9.6)**		**21 (16.4)**	**32 (12.0)**	
**> = 225**	**156 (26.9)**	**73 (6.1)**		**32 (25.0)**	**25 (9.4)**	
**Anaesthesia time (min)**			**<.001**			**<.001**
**< 190**	**41 (7.1)**	**353 (29.5)**		**11 (8.6)**	**92 (34.5)**	
**190–239.99**	**202 (34.8)**	**638 (53.3)**		**52 (40.6)**	**144 (53.9)**	
**> = 240**	**337 (58.1)**	**205 (17.2)**		**65 (50.8)**	**31 (11.6)**	
**Cardiopulmonary bypass time (min)**			**<.001**			**<.001**
**< 65**	**68 (11.7)**	**555 (46.4)**		**18 (14.1)**	**147 (55.1)**	
**65–94.99**	**184 (31.7)**	**519 (43.4)**		**40 (31.2)**	**101 (37.8)**	
**95–134.99**	**218 (37.6)**	**105 (8.8)**		**47 (36.7)**	**18 (6.7)**	
**> = 135**	**110 (19.0)**	**17 (1.4)**		**23 (18.0)**	**1 (0.4)**	
**Aortic occlusion time (min)**			**<.001**			**<.001**
**< 25**	**40 (6.9)**	**301 (25.1)**		**9 (7.1)**	**79 (29.6)**	
**25–54.99**	**239 (41.2)**	**770 (64.4)**		**51 (39.8)**	**164 (61.4)**	
**> = 55**	**301 (51.9)**	**125 (10.5)**		**68 (53.1)**	**24 (9.0)**	
**Operation time (min)**			**<.001**			**<.001**
**< 110**	**3 (0.5)**	**64 (5.4)**		**1 (0.8)**	**22 (8.2)**	
**110–179.99**	**248 (42.8)**	**910 (76.0)**		**61 (47.7)**	**220 (82.4)**	
**> = 180**	**329 (56.7)**	**222 (18.6)**		**66 (51.65)**	**25 (9.4)**	
**Intraoperative blood transfusion**			**<.001**			**<.001**
**YES**	**264 (45.5)**	**166 (13.9)**		**59 (46.1)**	**215 (80.5)**	
**NO**	**316 (54.5)**	**1030 (86.1)**		**69 (53.9)**	**52 (19.5)**	
**Preoperative total protein content (g/L)**			**0.03**			**0.038**
**< 59**	**99 (17.1)**	**158 (13.2)**		**18 (14.1)**	**20 (7.5)**	
**> = 59**	**481 (82.9)**	**1038 (86.8)**		**110 (85.9)**	**247 (92.5)**	
**Preoperative albumin (g/L)**			**0.509**			**0.486**
**< 39**	**15 (2.6)**	**25 (2.1)**		**5 (3.9)**	**7 (2.6)**	
**> = 39**	**565 (97.4)**	**1171 (97.9)**		**123 (96.1)**	**260 (97.4)**	

*PAH* Pulmonary hypertension, *PI* Pulmonary infection, *non-PI* Non- pulmonary infection

### Model development

The results showed that among 1776 patients, the number of candidate predictors was 13, so the sample size met the basic requirements to build the model. We also introduced a data set for external validation to verify the reliability of the model. No comparison to other relative risk prediction models was needed, as our model was the first model in the field.

### Statistical analysis

The measurement data in this study were all count data. Therefore, the count data were expressed as frequencies (percentages). The count data were analysed using the chi-square test. A multiple collinearity test was used to exclude confounding factors. Risk factor analysis was performed using univariate and multivariate logistic regression analyses. Data were not used in the multivariate logistic regression if the variance inflation factor (VIF) was greater than 10. Variables showing statistical significance in the univariate analysis were included in the multivariate logistic regression analysis, and the forward stepwise method was used to select the variables that were eventually included in the model [19].

Based on the collinearity diagnostics of the independent variables, we established an individualized nomogram prediction model of pulmonary infection following surgery for CHD in children.

Measures of calibration and discrimination assessed the predictive performance of the models. The prediction model was evaluated in terms of discrimination and calibration. The discrimination of the prediction model refers to its ability to distinguish between patients with pulmonary infection after surgery and patients with no pulmonary infection after surgery. A dichotomized outcome discrimination is most often assessed by calculating the area under the curve (AUC) of the receiver operating characteristic (ROC) curve. The AUC value is between 0.5 and 1.0 [20]. The closer the AUC value is to 1, the better the discrimination capacity the prediction model has. Generally, a prediction model with an AUC of 0.5–0.75 is considered acceptable, and an AUC > 0.75 indicates that the model shows excellent discrimination [19].

The statistical analyses were performed using SPSS version 24 (IBM, Armonk, NY, U.S.A) and RStudio software (U.S.A).

## Results

### Patient demographics

In this study, a total of 2171 patients were enrolled, with 1776 in the development group and 395 in the validation group. In the development group, 580 patients presented pulmonary infections and 1196 patients did not develop pulmonary infections. In the validation group, 128 patients presented pulmonary infections and 267 patients did not develop pulmonary infections. Age and weight were significantly lower, RACHS-1 scores were significantly higher, preoperative hospital stay was significantly longer and intraoperative blood transfusion occurred more often in the pulmonary infection group than in the non-pulmonary infection group. In the non-pulmonary infection group, anaesthesia time, cardiopulmonary bypass time, aortic occlusion time and operation time were significantly shorter than those in the pulmonary infection group (Table 1).

### Nomogram development

The univariate analysis of the development group showed that the statistically significant risk factors were age, weight, RACHS-1 score, preoperative hospital stay, anaesthesia time, cardiopulmonary bypass time, aortic occlusion time and intraoperative blood transfusion (*P* < 0.05), whereas sex, pulmonary hypertension, preoperative total protein content and preoperative albumin were not related to pulmonary infection (Table 2). The statistically significant variables from the univariate analysis were included in the non-conditional binary multivariate logistic regression. The six factors of age, weight, RACHS-1 score, preoperative hospital stay, cardiopulmonary bypass time and intraoperative blood transfusion were independent risk factors of pulmonary infection after surgery for CHD (Table 2). We conducted collinearity diagnostics for the above independent risk factors, and the variance inflation factors VIFs were 2.869,2.877,1.394,1.100,1.535 and 1.243 respectively, suggesting that there was no multiple collinearity among the six independent risk factors.

**Table 2:** Univariate and multivariate logistic regression models for the development group

Factor	Subgroup	Univariate analysis		Multivariate analysis	
		OR (95%CI)	***P*** Value	OR (95%CI)	***P*** Value
**Sex**		**0.97 (0.797–1.186)**	**0.782**	**NA**	
**Age**	**< 6**				
	**6–11.99**	**0.58 (0.41–0.86)**	**0.006**	**0.71 (0.43–1.17)**	**0.175**
	**12–23.99**	**0.34 (0.23–0.48)**	**< 0.001**	**0.37 (0.21–0.66)**	**0.001**
	**> = 24**	**0.11 (0.08–0.16)**	**< 0.001**	**0.20 (0.10–0.39)**	**< 0.001**
**Weight**	**< 5**				
	**5–7.99**	**0.32 (0.19–0.54)**	**< 0.001**	**0.54 (0.27–1.06)**	**0.075**
	**8–13.99**	**0.15 (0.09–0.25)**	**< 0.001**	**0.42 (0.19–0.91)**	**< 0.001**
	**> = 14**	**0.05 (0.03–0.08)**	**< 0.001**	**0.13 (0.05–0.34)**	**< 0.001**
**PAH**		**0.89 (0.73–1.09)**	**0.251**	**NA**	
**RACHS-1**		**13.17 (10.06–17.24)**	**< 0.001**	**6.94 (4.86–9.90)**	**< 0.001**
**Preoperative hospital stay**	**< 125**				
	**125–174.99**	**2.02 (1.57–2.60)**	**< 0.001**	**1.80 (1.30–2.50)**	**< 0.001**
	**175–224.99**	**3.19 (2.30–4.43)**	**< 0.001**	**2.37 (1.52–3.70)**	**< 0.001**
	**> = 225**	**8.81 (6.34–12.23)**	**< 0.001**	**6.71 (4.33–10.40)**	**< 0.001**
**Anaesthesia time**	**< 190**			**NA**	
	**190–239.99**	**2.73 (1.90–3.91)**	**< 0.001**	**NA**	
	**> = 240**	**14.154 (9.81–20.43)**	**< 0.001**	**NA**	
**Cardiopulmonary bypass time**	**< 65**				
	**65–94.99**	**2.89 (2.14–3.92)**	**< 0.001**	**1.68 (1.15–2.47)**	**< 0.001**
	**95–134.99**	**16.95 (12.03–23.88)**	**< 0.001**	**3.92 (2.34–6.56)**	**< 0.001**
	**> = 135**	**52.81 (29.88–93.34)**	**< 0.001**	**7.76 (3.56–16.95)**	**< 0.001**
**Aortic occlusion time**	**< 25**			**NA**	
	**25–54.99**	**2.34 (1.63–3.35)**	**< 0.001**	**NA**	
	**> = 55**	**18.12 (12.27–26.77)**	**< 0.001**	**NA**	
**Operation time**	**< 110**			**NA**	
	**110–179.99**	**5.81 (1.81–18.66)**	**< 0.001**	**NA**	
	**> = 180**	**31.62 (9.81–101.89)**	**< 0.001**	**NA**	
**Intraoperative blood transfusion**		**5.18 (4.11–6.53)**	**< 0.001**	**1.67 (1.20–2.32)**	**0.002**
**Preoperative total protein content**		**0.74 (0.56–0.97)**	**0.03**	**NA**	
**Preoperative albumin**		**0.80 (0.42–1.54)**	**0.51**	**NA**	

**Fig. 1:**
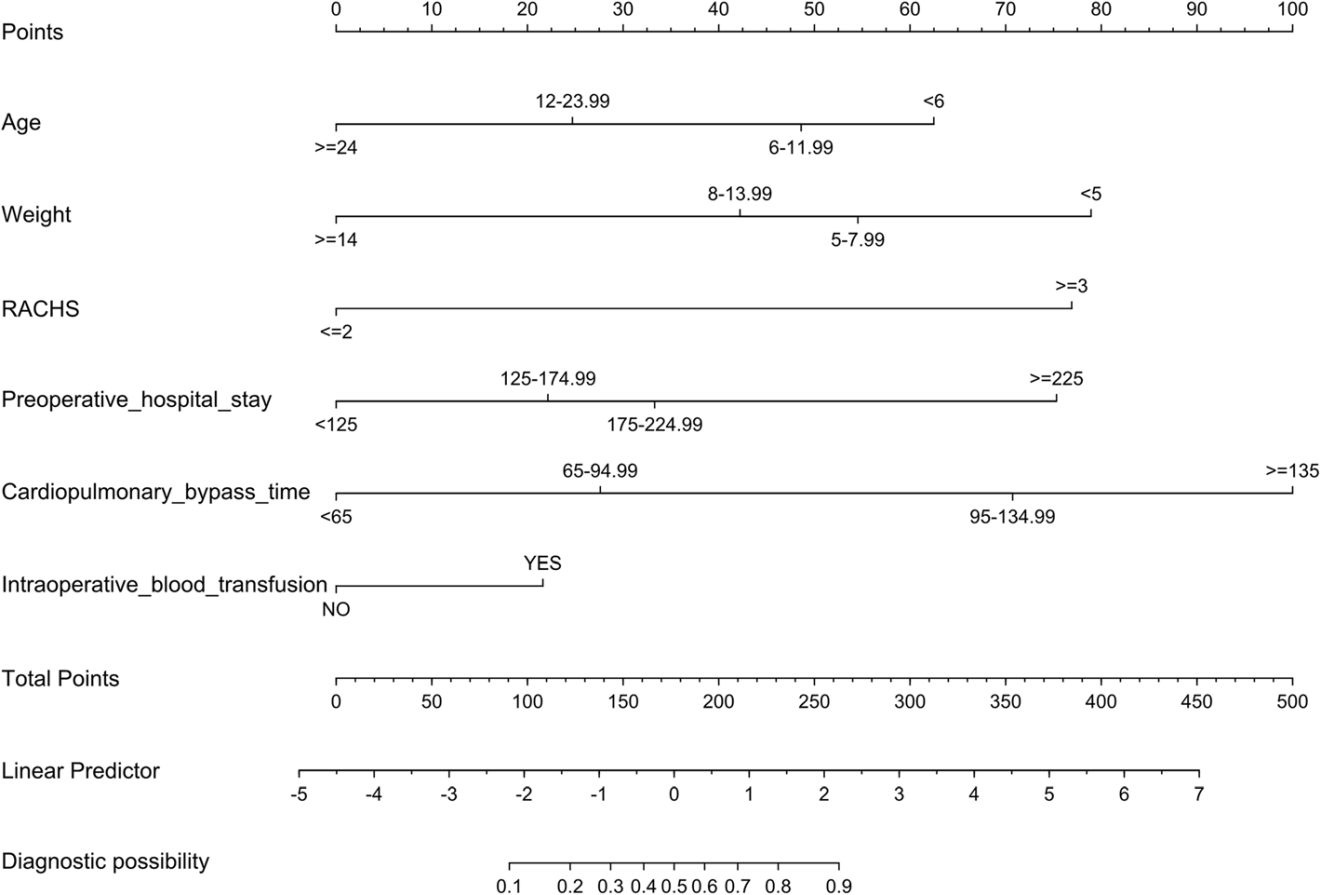
Nomogram to predict the probability of pulmonary infection after cardiopulmonary bypass surgery. To estimate the probability of pulmonary infection after cardiopulmonary bypass surgery, the value of each factor is acquired on each variable axis, then a straight line is drawn upward to determine the points. The sum of these 6 numbers is located on the Total Points axis and a line is drawn downward to the Diagnostic Possibility axes to determine the likelihood of pulmonary infection after cardiopulmonary bypass surgery

Based on the logistic multivariate regression analysis, the six independent risk factors were included in the prediction model. We then establish an individualized nomogram prediction model of pulmonary infection after surgery (Fig. 1).

### Nomogram validation

**Fig. 2:**
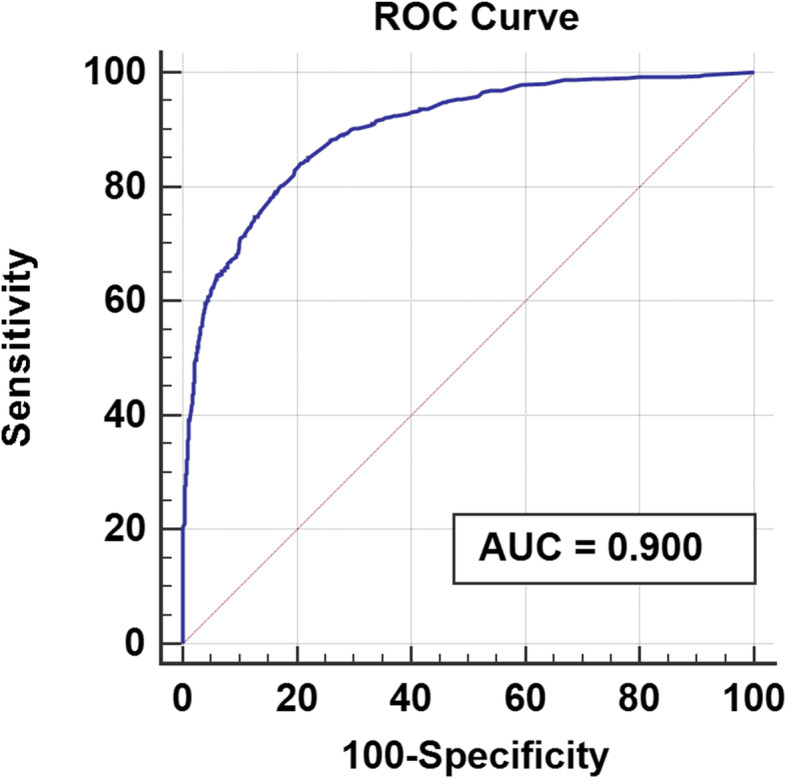
ROC curves for Development group

**Fig. 3:**
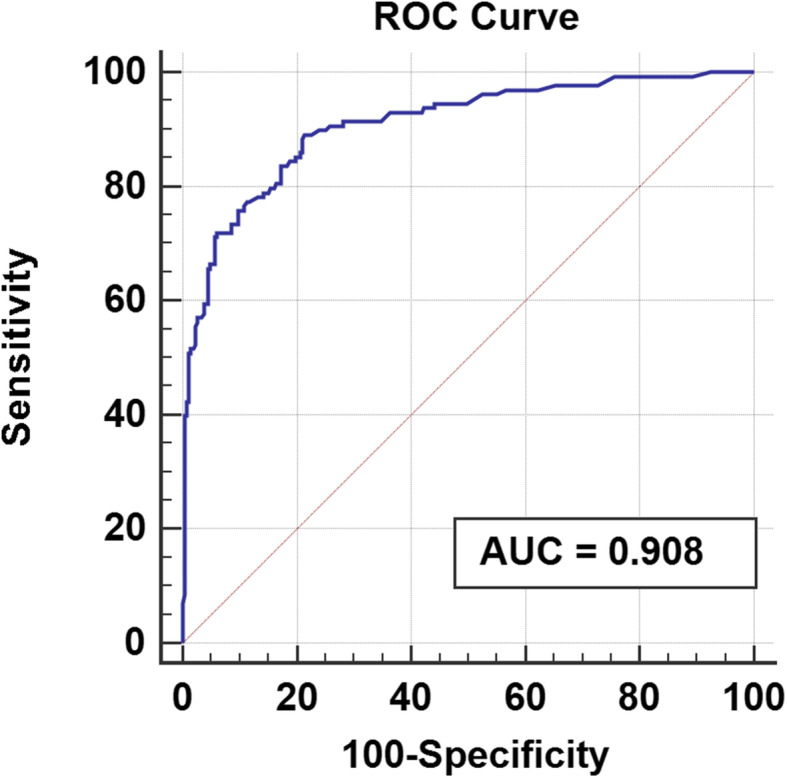
ROC curves for Validation group

**Fig. 4:**
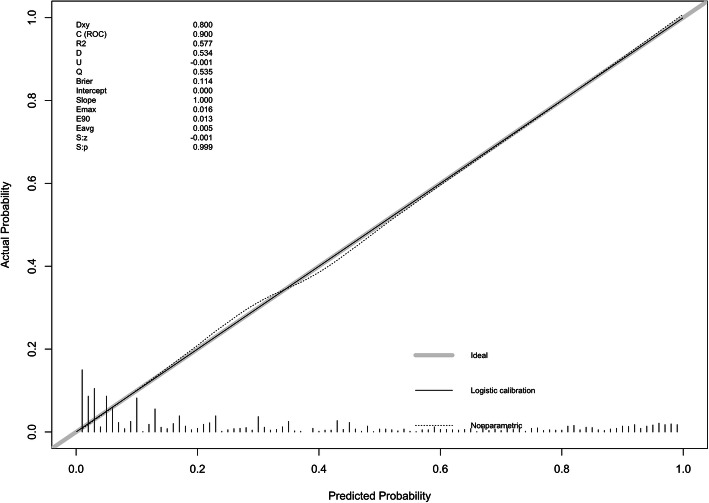
Calibration plots of the nomogram for the probability of pulmonary infection after cardiopulmonary bypass surgery. (Development group)

**Fig. 5:**
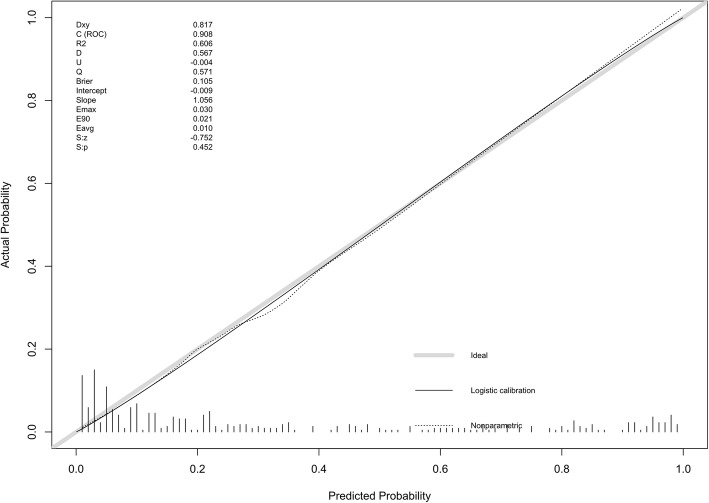
Calibration plots of the nomogram for the probability of pulmonary infection after cardiopulmonary bypass surgery. (Validation group)

The validation of the model was based on discrimination and calibration. We drew the ROC curves of the predicted probability (Figs. 2, 3) and calculated the AUCs for the development and validation groups [20]. The ROC curve was used to compare the AUC values of the six independent risk factors from the nomogram and multivariate analysis (Table 3); the differences were statistically significant (*P* < 0.05). The AUC values were 0.900 and 0.908 for the development group and the validation group. The *P*-values for the calibration tests for the two groups were 0.999 and 0.452 respectively (Figs. 4, 5). Therefore the predicted probability of the model was consistent with the actual probability [21].

**Table 3:** The AUCs of the ROC curves for the nomogram and variables from the logistic regression model for the development group and validation group

	Development group			Validation group		
	AUC	95%CI	***P*** value	AUC	95%CI	***P*** value
**Nomogram variable**	**0.900**	**0.885–0.913**	**< 0.001**	**0.908**	**0.876–0.935**	**< 0.001**
**Age**	**0.707**	**0.685–0.728**	**< 0.001**	**NA**	**NA**	**NA**
**Weight**	**0.700**	**0.678–0.721**	**< 0.001**	**NA**	**NA**	**NA**
**RACHS-1**	**0.721**	**0.700–0.742**	**< 0.001**	**NA**	**NA**	**NA**
**Preoperative hospital stay**	**0.683**	**0.661–0.704**	**< 0.001**	**NA**	**NA**	**NA**
**Cardiopulmonary bypass time**	**0.786**	**0.766–0.804**	**< 0.001**	**NA**	**NA**	**NA**
**Intraoperative blood transfusion**	**0.658**	**0.636–0.680**	**< 0.001**	**NA**	**NA**	**NA**

## Discussion

Pulmonary infection is a major cause of morbidity and mortality in paediatric patients undergoing cardiac surgery [2]. And patients with CHD have unique characteristics, ischemia-reperfusion injury, low temperature and surgical trauma can cause transient immunosuppression in the body [5, 6]. Therefor, the occurrence of pulmonary infection after cardiac surgery is reported to be high at home and abroad [2, 3]. Our study showed that the incidence of pulmonary infection in our centre was approximately 30%. For a surgeon, controlling perioperative pulmonary infection is a very serious consideration. But we donundefinedt know which patients be at high risk of pulmonary infection after surgery. Often, patients with pulmonary infections were found when the symptoms are serious. Therefore, it is very important to identify patients with a high-risk of pulmonary infection after CHD surgery. Patients with a high-risk of infections after surgery should be identified early so that medical staff can focus on monitoring and care, and if possible, administe interventional treatments in advance [22]. In our study, we identified risk factors for pulmonary infection and created a model that can be used to estimate a patient’s pulmonary infection risk. We validated the model by using another data set for external validation and showed that it had good discrimination. The clinical tool can help us assess the risk of pulmonary infection for the patients following the end of the procedure.

Previous studies have evaluated risk factors for specific types of postoperative infections. Cardiopulmonary bypass time, an increased duration of surgery, a prolonged preoperative stay, young age and low body weight increased the risk of infection. Prolonged cardiopulmonary bypass can easily lead to the decrease of immune function and the disorder of water electrolyte and acid-base balance. The cardiopulmonary function and immune function of children with CHD are poor, and often need longer cardiopulmonary bypass support, which will significantly increase the burden of the body, further decrease the immune function of children, resulting in long cardiopulmonary bypass time become a risk factor for the occurrence of pulmonary infection after congenital heart disease. With the growth of children, the development of their own respiratory tract and the establishment of immune function, so pulmonary infection is more likely to occur in children of young age and low body weight [2, 3, 7, 18, 23–26] .The review by Dresbach et al. [27] reported the central venous catheter (CVC) indwelling time was an independent risk factor for infection. Costello et al. [28] reported that the transfusion of blood products was an independent risk factor for infection. However, none of the previous studies developed a model considering preoperative and operative factors that can be clinically applied to predict the risk of pulmonary infection in patients who treated in the CICU after surgery [14]. In this study, the factors that were significantly associated with pulmonary infection in the multivariable analysis were largely similar to previously identified risk factors. The factor that accounted for the greatest increase in risk was high complexity. Anaesthesia time, aortic occlusion time and operation time were associated with pulmonary infection in the univariate analysis, but did not remain risk factors according to the multivariable analysis, suggesting that these factors may be reflected in cardiopulmonary bypass time. In our study, sex, pulmonary hypertension, preoperative albumin content, and intraoperative albumin content were not associated with pulmonary infection in the univariate analysis. Some previous studies reported that postoperative ventilator time was a risk factor [14]. Our clinical tool was created to be used at the end of the surgery, so we did not include this candidate variable in our study.

Based on the characteristics of patients with CHD in our region, we successfully developed a risk prediction model based on age, weight, RACHS-1 score, preoperative hospital stay, cardiopulmonary bypass time and intraoperative blood transfusion. An objective evaluation of a model is necessary, and a valuable model can be characterized by the 2 related properties of discrimination and calibration [19, 20]. The model was regarded as clearly useful according to the discrimination analysis because the AUC was more than 0.75. The calibration of the prediction model was perfect in both groups. The discrimination and calibration results indicated that the model results were similar to the actual situation [29]. We constructed a nomogram to reduce multiple statistical predictive models into a single numerical estimate of the probability of an event [30]. Doctors can use the nomogram to easily assess the possibility of pulmonary infection following surgery for CHD.

In our study, all patients had no pulmonary infection before surgery, and the first generation of cephalosporins were used to prevent infection before surgery. After the operation, they were sent to the CICU for further treatment. In the CICU, all patients were all isolated and treated individually by medical staff to minimize exposure to external sources of infection exposure and other factors. Patients with pulmonary infection after surgery were diagnosed, we would adopt strong airway management and antibiotic treatment schemes. However, we attempted to assess our patients after operation as soon as they were admitted to the CICU, in order to assess the possibility of pulmonary infection following surgery for CHD. For patients with high possibility of pulmonary infection, we would conduct more intensive care, and adopt strong airway management, such as emphasizing physical support treatment of the respiratory tract, moisturizing sputum, and timely clearing of airway secretions, etc. Such patients need to pay attention to the role of auxiliary therapies such as oxygen therapy and sedation. Previous studies have shown that gram-negative bacilli are the most common pathogens in pulmonary infection after cardiopulmonary bypass surgery [18], patients with a high-risk of infections after surgery were screened out by our clinical tool, we speculate that the use of antibiotics against gram-negative bacilli may achieve ideal results for these patients.

The strengths of this study are that this was the first risk prediction model for pulmonary infection following cardiopulmonary bypass surgery for CHD in children in our region, and the developed model can be easily applied in clinical situations. However, there are some limitations to this study. First, it was a retrospective study, that could not avoid selection bias. However, we strictly set the inclusion criteria and collected relatively adequate clinical samples so that our clinical tool the truly reflected the actual condition of occurrence. Second, the study was a single-centre study. Although we used patient samples from different periods to validate the model, we still need evidence from other centres for validation. Multicentre studies with large sample should be developed in the future. Therefore, in the follow-up research, we will persuade other medical centres to join this research project and will provide the appropriate clinical data to conduct an in-depth assessment and validation of the prediction model.

## Conclusions

An individualized nomogram prediction model was developed to evaluate pulmonary infection in patients after surgery. We can accurately predict the risk of pulmonary infection in patients after surgery with this prediction model. For high-risk patients, after surgery, targeted interventions to reduce the risk of pulmonary infection should be applied.

## Data Availability

The datasets used and analyzed during this study are available from the corresponding author on reasonable request.

## Abbreviations

CHD: Congenital heart disease
CICU: Cardiac intensive care unit
ROC: Receiver operating characteristic curve
HAIs: Hospital-acquired infections
CVC: Central venous catheter
